# Virtual patients in medical education: a bibliometric analysis based on the wos core collection and scopus databases

**DOI:** 10.3389/fmed.2026.1904001

**Published:** 2026-07-30

**Authors:** Yinglan Ye, Hairong Tao, Siqi Huang, Meixia Zou, Yiming Chen, Peifeng Shen, Man Hao, Chunlong Liu

**Affiliations:** Clinical Medical College of Acupuncture Moxibustion and Rehabilitation, Guangzhou University of Chinese Medicine, Guangzhou, Guangdong, China

**Keywords:** bibliometric analysis, citespace, generative artificial intelligence, large language models, medical education, virtual patients, VOSviewer

## Abstract

**Background and objective:**

Virtual patients have become an integral component of medical education, offering medical students realistic, repeatable, and interactive learning environments that effectively foster the development of clinical competencies. Moreover, the rapid evolution of generative artificial intelligence and large language model technologies has further accelerated the integration of virtual patients into medical education, making it a prominent area of contemporary educational innovation. To provide a comprehensive overview of the research landscape and emerging trends in this domain, we performed a bibliometric and knowledge-mapping analysis of the literature on virtual patients in medical education.

**Methods:**

Publications published between 2006 and 2025 were retrieved from the Web of Science Core Collection and Scopus databases, including only English articles and review articles. CiteSpace and VOSviewer were used to perform bibliometric and knowledge-mapping analyses of collaboration networks, co-citation patterns, and keyword evolution.

**Result:**

A total of 2,347 publications showed a consistent upward trend. The United States led contributions, followed by the United Kingdom and Germany, with extensive collaborations between North America and Europe. *Medical Education* was the most influential journal; Karolinska Institutet was the most productive institution and Cook, DA. was the most prolific author. Frequent keywords included “medical education,” “simulation,” “clinical competence,” “virtual reality,” and “patient simulation.” The most cited article confirmed virtual patients outperform no intervention, providing an evidence base for their adoption in medical education.

**Conclusions:**

This bibliometric analysis highlights the sustained growth and evolving research landscape of virtual patients in medical education. The findings indicate that recent research has increasingly focused on the integration of generative artificial intelligence and large language models into virtual patient systems. These findings provide a quantitative evidence base for future research priorities and the continued development of virtual patient-based medical education.

## 1 Introduction

Virtual patients (VPs) constitute a pivotal technology-driven educational modality within modern medical education. Through computer-based, interactive simulations of clinical encounters, VPs provide learners with opportunities to develop and refine clinical reasoning, communication, and decision-making skills within a safe and controlled learning environment (1–3). Over the past two decades, the integration of VPs into medical curricula has increased steadily, driven by the growing demand for scalable, standardized, and reproducible approaches to clinical training (4). Evidence consistently supports the effectiveness of VPs in improving clinical competence, with learning outcomes comparable to, and in some cases superior to, those achieved through traditional educational methods (5). Cook et al. (6) argued that VPs not only promote analytical reasoning but also support the development of intuitive, experience-based clinical thinking. Subsequent studies have further confirmed the educational value of VPs in clinical skills training. For example, Consorti et al. (7) and Saleh et al. (8) demonstrated that VP-based learning enhances learners' practical competencies while reducing the patient safety risks associated with direct clinical practice, highlighting the value of VPs as effective tools for both teaching and assessment. More recently, advances in generative artificial intelligence (GenAI), particularly the emergence of large language models (LLMs) such as ChatGPT, have facilitated the development of next-generation VPs capable of responding dynamically, creating increasingly realistic, adaptive, and immersive learning experiences (9).

Research on VPs has expanded steadily over the past two decades, driven by evolving educational needs and continuous technological advances. More recently, two major developments—the COVID-19 pandemic (2019–2021) and the emergence of GenAI and LLMs (2022–present)—have influenced the development of VPs research (10, 11). The COVID-19 pandemic has reshaped research priorities and demands, while the development of GenAI, particularly the emergence of LLMs, has expanded the technological capabilities of VP systems. Specifically, The pandemic necessitated an unprecedented shift toward remote learning, significantly reducing access to bedside teaching and clinical training opportunities. Consequently, virtual patients emerged as important educational tools for ensuring continuity in clinical education and skills development (12). Following this period of increased digital adoption, advances in GenAI technologies have initiated a new phase in the evolution of VPs. Powered by LLMs and natural language processing, next-generation virtual patients are capable of engaging in real-time, adaptive, and context-aware interactions with learners (13, 14). These advances enable automated case generation, personalized feedback, and increasingly authentic clinical conversations, substantially improving the realism, scalability, and educational value of VP-based learning environments (15, 16).

Bibliometric analysis is an interdisciplinary quantitative method used specifically for studying the literature related to a particular field. This statistical tool allows for building connections between published works, uncovering research hotspots in academic fields, and quantitatively tracking the development trends of research topics (17). Compared to traditional reviews, bibliometric analysis has the advantage of objectively presenting the internal conceptual structure and potential connections of a large volume of publications. Accordingly, this study applies bibliometric methods to quantitatively examine the development of VP research in medical education, with a particular focus on the major impacts of the COVID-19 pandemic and recent advances in GenAI. Specifically, we analyze influential publications, citation patterns, and thematic evolution within the field, while identifying emerging technologies that are shaping the next generation of virtual patients. By mapping the intellectual structure, research hotspots, collaboration patterns, and thematic evolution of VP research, this bibliometric study provides quantitative evidence to inform future research, educational practice, and policy development.

## 2 Methods and materials

### 2.1 Data sources and search strategies

Web of Science (WOS) is a high-quality multidisciplinary database that includes over 9,500 core journals and features powerful citation analysis capabilities (18). Scopus was selected for its comprehensive coverage and diverse literature. Additionally, it ranks as the world's largest standalone abstract and indexing database, boasting comprehensive literature retrieval resources and the most extensive searchable citation repository (19).

**Figure 1:**
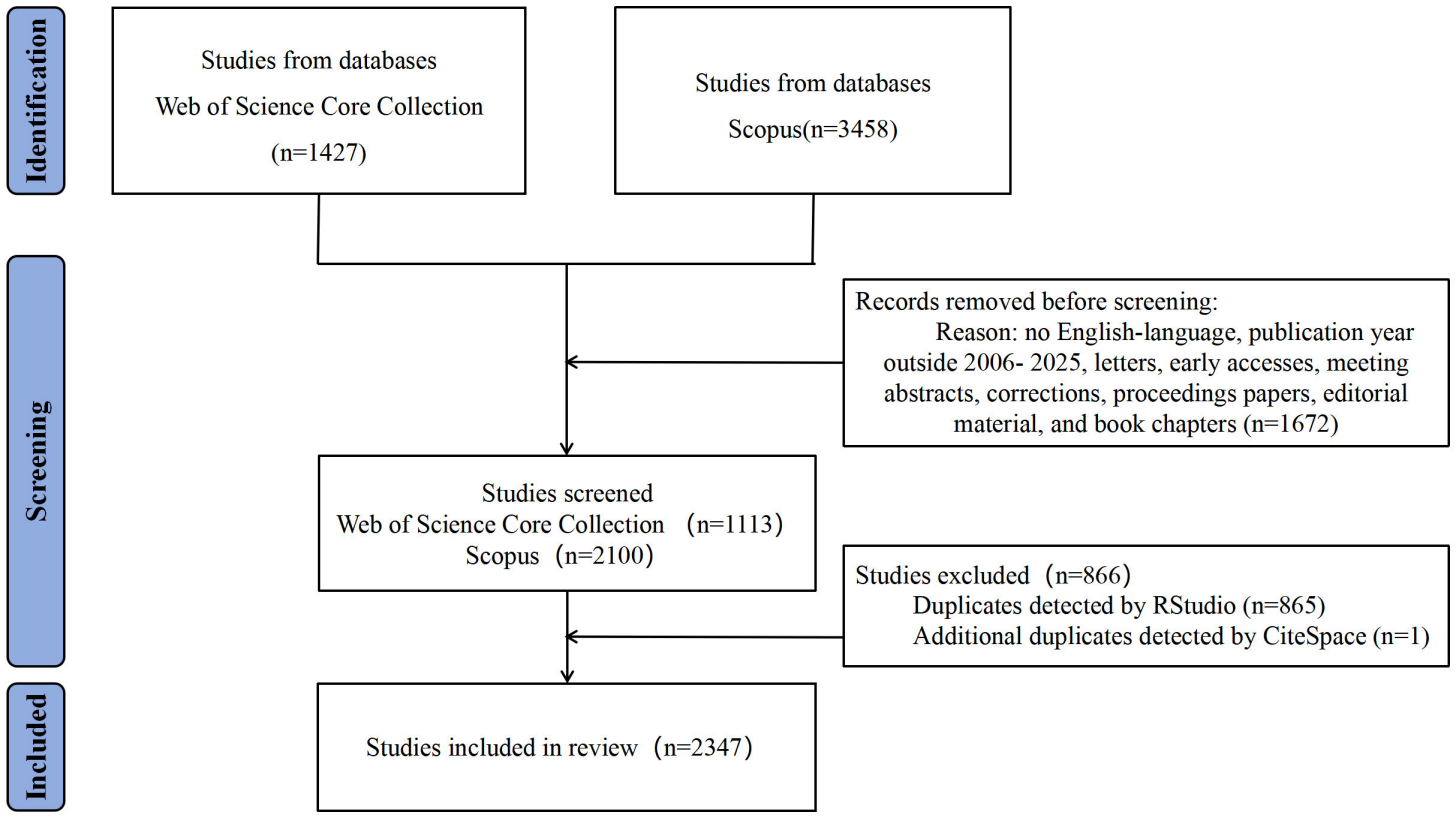
Database search strategy based for the literature on virtual patients in medical education.

The literature search was conducted on February 19, 2026, by a single researcher. Searches of both the WOS Core Collection and Scopus databases were completed on the same day to minimize potential bias arising from database updates. This study retrieved English articles and reviews from 2006 to 2025 (up to December 31) concerning VP research in medical education. A total of 1,427 records were obtained from WOS Core Collection, and 3,458 records were retrieved from Scopus, with an initial combined pool of 4,885 records. Peliminary filtering was performed according to preset eligibility criteria: non-English articles, publications outside the 2006–2025 time range, letters, early access articles, meeting abstracts, corrections, conference proceedings, editorial materials and book chapters were excluded. After this filtering step, 1,672 records were removed, leaving 3,213 records (1,113 from Web of Science, 2,100 from Scopus). Next, duplicates were identified and removed using two tools: RStudio identified 865 duplicate records, and CiteSpace supplemented the detection of 1 extra duplicate, resulting in 866 duplicated references removed in total. After deduplication, 2,347 unique articles were finally included for subsequent bibliometric analysis. The complete search strings applied in two databases are available in Supplementary Table S1. The whole screening workflow is visually presented in the Figure 1.

To ensure the validity of the bibliometric findings, we conducted a secondary manual verification of the visualized results. As *Patient Education and Counseling*, the third most productive journal in our analysis, is widely recognized as an interdisciplinary journal focusing on patient education, counseling, and healthcare communication, we further assessed the relevance of its included publications. Two researchers independently screened the titles and abstracts of all retrieved articles to determine their eligibility for this study. Any disagreements were resolved through full-text review by a third researcher. This verification confirmed that the included articles primarily focused on patient simulation–based educational approaches for medical students and healthcare trainees, particularly the development of patient-centered competencies such as communication skills, empathy, and breaking bad news, rather than patient education for the general public. Therefore, we concluded that the publications from this journal were relevant to the scope of the present study and were appropriately retained in the analysis.

### 2.2 Analysis tools

This study utilized four analytical tools for data processing and visual mapping: CiteSpace (6.4.R1), VOSviewer (1.6.18), Bibliometrix R package (version 4.5.2), and Microsoft Office Excel (Office16).

CiteSpace, a Java-based bibliometric visualization tool, supports knowledge network visualization, time-slicing analysis, and the identification of research hotspots and frontiers. In this study, it was used for data deduplication, format standardization, and the construction of co-citation, collaboration, and keyword co-occurrence networks. Indicators including burst terms, cluster labels, and betweenness centrality were applied to identify research hotspots and key nodes (20–22).

Similarly, VOSviewer, also Java-based, employs models such as co-word, co-citation, collaboration, and coupling to generate density maps, overlay maps, and cluster maps (23). It utilizes VOS mapping and modularity algorithms to present the structure of research themes. Node size is proportional to frequency, citation counts, or link strength, while color indicates the number of themes. The thicker the lines, the stronger the co-occurrence or co-citation relationships (24).

Bibliometrix is an open-source R package for bibliometric and science mapping analysis that supports database merging, data preprocessing, and duplicate record removal prior to bibliometric analysis (25).

Microsoft Office Excel was used to generate publication quantity and trend charts. Two common indicators used to assess the strength of variable relationships and predictive ability are the indicator and *R*^2^. A positive coefficient indicates a positive correlation and an upward trend, while an *R*^2^ value closer to 1 indicates a better fit (26).

A citation burst indicates a rapid increase in frequency for a node within a specified timeframe, reflecting the dynamic changes and frontier issues of the research field (27, 28). If a node exhibits a sustained burst, it can suggest continued upward trajectory and future research trends (29). The burst intensity and duration are central elements in this detection, with keywords that burst until 2025 indicating current hotspots.

Price's Law, proposed by scientometrics scholar Derek J. de Solla Price, was applied to evaluate literature productivity and author influence. The core author threshold was calculated using the formula M ≈ 0.749 × √Nmax, where M represents the minimum number of publications required for core authorship and Nmax denotes the publication count of the most prolific author. Authors with publications exceeding M were defined as core authors. A core author group was considered established when its cumulative publications accounted for more than 50% of the total literature output, reflecting the developmental maturity of the field (30).

## 3 Results

### 3.1 General characteristics and trends of publications

**Figure 2:**
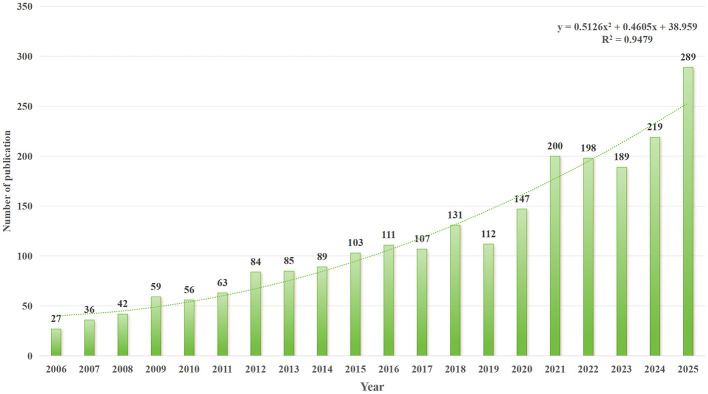
Publication output, temporal trends and model fitting of VP researchs in medical education.

Among the 2,347 articles included in the analysis, the annual publication volume and corresponding trend are depicted in Figure 2 From 2006 to 2025, research concerning VPs in medical education has demonstrated a consistent upward trajectory, attaining new peaks particularly within the past 5 years. During the pre-pandemic period (2006–2018), the annual number of publications in this field increased steadily from 27 to 137, reflecting a gradual and stable expansion. The annual publication output declined from 137 in 2018 to 112 in 2019. Subsequently, during the COVID-19 pandemic (2019–2021), research output increased sharply, reaching 200 publications in 2021, representing a 78.57% increase compared to 2019 (112 publications). Although the annual number of publications declined slightly to 198 in 2022 and 189 in 2023, research output in both years remained substantially higher than that of any year before 2020. Publication output subsequently resumed its upward trend, increasing to 219 in 2024 and reaching a peak of 289 in 2025. Overall, the number of publications grew by 45.95% between 2022 and 2025, indicating that the growth rate of research output accelerated during the COVID-19 pandemic and continued to increase in the post-pandemic period. The number of publications increased from 27 articles in 2006 to 289 articles in 2025, representing a 970% growth. A polynomial regression model fitted to the annual data yields an *R*^2^ value of 0.9479, which further substantiates the trend of escalating research output in this field.

### 3.2 Analysis of countries/regions and institutions

**Figure 3:**
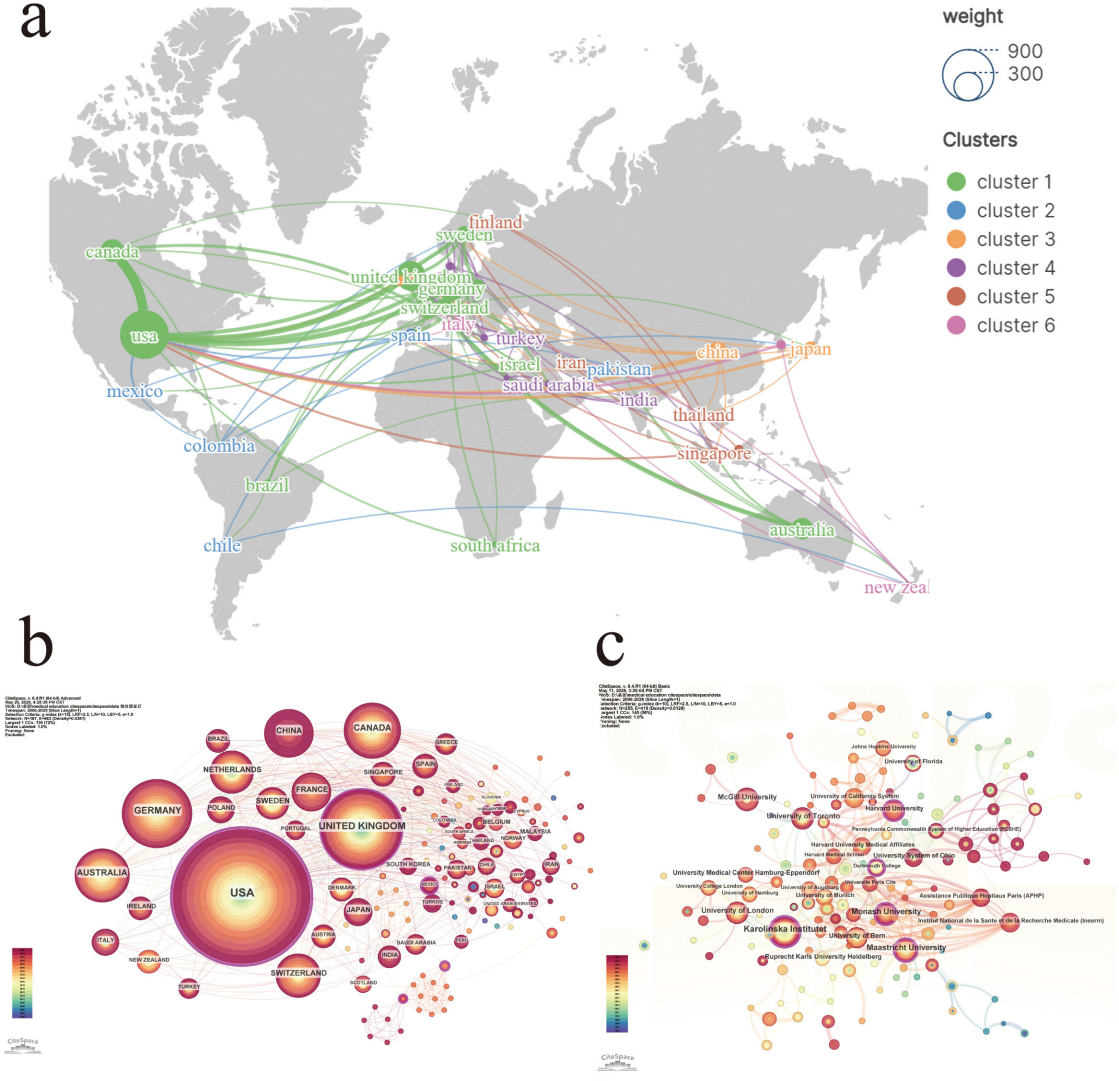
The co-occurrence analysis profile of the countries/regions, and institutions. **(a)** International collaboration network. Node size represents publication volume (weight: 300–900), lines stand for cooperative relationships, the thicker the line, the higher the frequency of collaboration between the two countries, and colors denote different research communities. **(b)** CiteSpace network of research countries/regions. **(c)** CiteSpace network of research institutions. In figure **(b, c)**, node rings show publication years, widths indicate annual output, and links represent co-occurrence ties, color gradients mark each term's initial emergence.

Publication counts by country were used to analyze contributions in this field. Table 1 lists the top 10 countries/regions and institutions by publication volume, while Figure 3a displays their global distribution. Data were imported into CiteSpace for visualization analysis (Figure 3b, c).

**Table 1:** Top 10 most productive countries/regions and institutions.

Rank	Country	Count (%)	Centrality	Institution	Count (%)	Centrality
1	USA	763 (32.51%)	0.56	Karolinska Institutet	49 (2.08%)	0.12
2	United Kingdom	282 (12.01%)	0.15	Maastricht University	40 (1.70%)	0.14
3	Germany	234 (9.97%)	0.02	Monash University	37 (1.57%)	0.10
4	Canada	161 (6.85%)	0.06	University of Toronto	27 (1.15%)	0.07
5	Australia	146 (6.22%)	0.04	University of London	26 (1.10%)	0.06
6	China	115 (4.89%)	0.04	McGill University	25 (1.06%)	0.03
7	Netherlands	90 (3.83%)	0.02	University of Bern	23 (0.97%)	0.03
8	Switzerland	89 (3.79%)	0.02	Harvard University	21 (0.89%)	0.10
9	France	79 (3.36%)	0.04	University System of Ohio	20 (0.85%)	0.06
10	Sweden	70 (2.98%)	0.02	University Medical Center Hamburg-Eppendorf	18 (0.76%)	0.00

According to Figure 3a, the results show that the United States has the highest node weight, serving as a central hub in the network with cooperative relationships with the vast majority of countries. Cluster analysis identified six major cooperative communities: the cluster centered on the United States, the United Kingdom, and Germany is the largest and exhibits the strongest internal cooperation; Latin America, Asia, Europe, and other regions have each formed their own cooperative subnetworks, demonstrating clear regional cooperation preferences. Countries such as the United States and the United Kingdom engage in frequent cooperation with multiple clusters simultaneously, acting as key nodes for cross-regional cooperation.

Numerous institutions have contributed to research on VPs in medical education. The Karolinska Institutet leads in publication volume with 49 articles (2.08%), whereas Maastricht University exhibits the highest centrality (0.14). The top 10 institutions are primarily located in Europe and North America, reflecting the strong research capabilities in this field in those regions. Figure 3c illustrates the collaborative network among these institutions.

### 3.3 Analysis of authors and co-cited authors

Supplementary Table S2 lists the top 10 authors by publication volume. Among them, Harendza, S. has the highest number of publications (22 articles). Price's Law (M ≈ 0.749 × √Nmax) was used to determine core authors. The maximum individual publication number Nmax = 22, yielding a threshold of M ≈ 3. Authors with over three publications were regarded as core authors. Core authors produced 1,487 papers, taking up 63.3% of total publications (1,487/2,347). The proportion above 50% demonstrates that the research field has developed into a mature academic system. Supplementary Figure S1a illustrates the author collaboration network, where node size is proportional to publication volume.

Supplementary Figure S1b depicts the author co-citation network, highlighting authors with 30 or more citations as core. Node size represents influence, and line thickness indicates co-citation strength. Cook, DA, has the highest number of citations. Supplementary Table S3 lists the top 10 authors by co-citation count.

### 3.4 Analysis of journals

**Figure 4:**
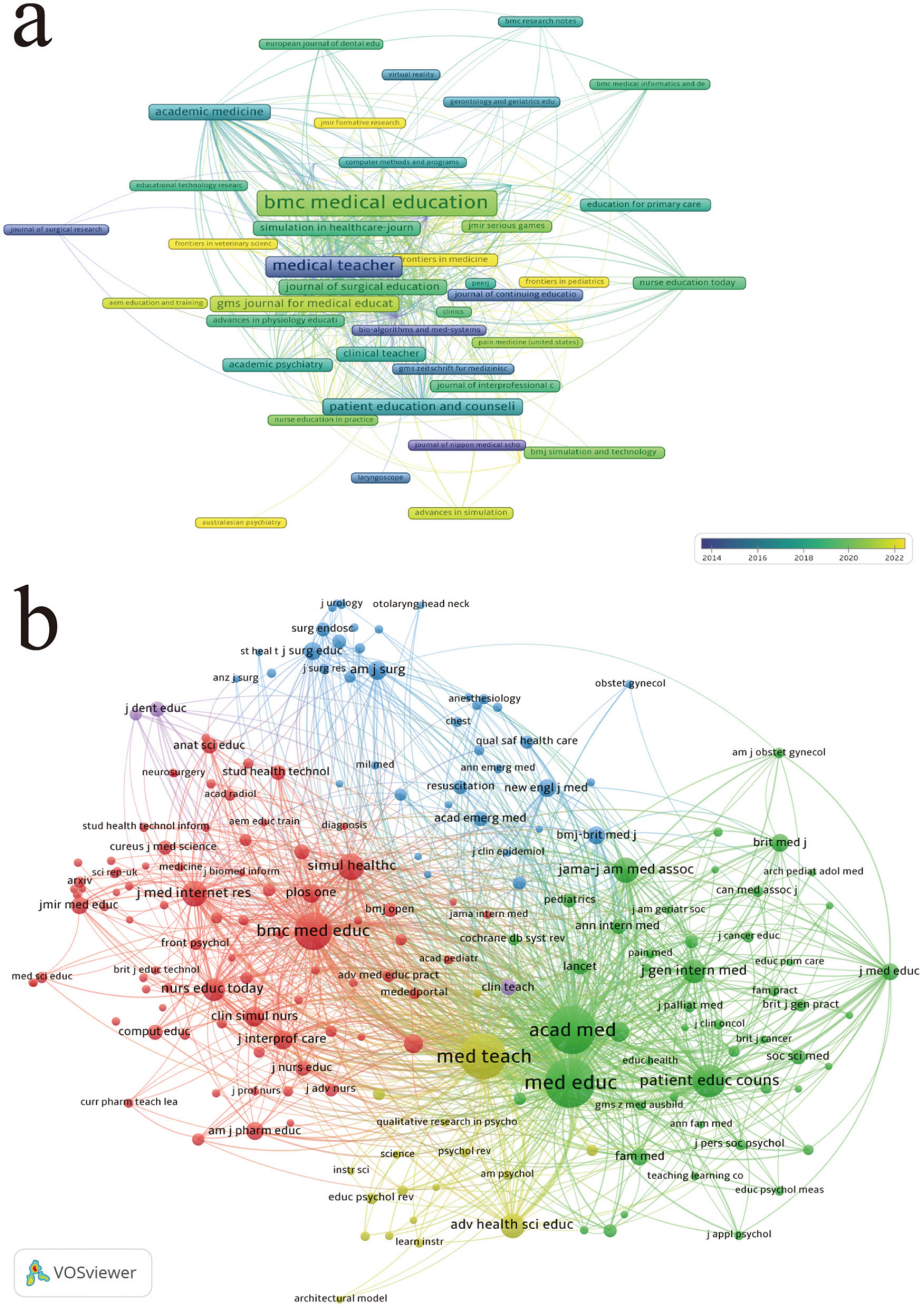
VOSviewer generated a network visualization of the journal co-citation analysis. **(a)** Analysis of collaborative network visualization of journals in VOSviewer. Node size denotes frequency of the journal's appearance, line thickness indicates inter-journal academic relevance, and node color reflects the peak co-citation year. **(b)** VOSviewer-generated network visualization map of journal co-citation analysis. Node size represents the co-citation frequency of journals, line strength reflects the co-citation relationships between journals.

Between 2006 and 2025, a total of 2,347 articles on VPs in medical education were published across 895 journals. Tables 2, 3 lists the 10 most prolific sources and journals ranked by publication volume, along with their 2024 impact factor (IF). The top 10 journals published a total of 671 papers. Among these, *BMC Medical Education* had the highest number of publications. The journal co-citation network map (Figure 4a) visualizes the intellectual base structure of research on VPs in medical education. The results show that *BMC Medical Education* and *Medical Teacher* are the core hub journals in this field. Temporally, high co-citations of traditional medical education journals (e.g., *Medical Teacher, Academic Medicine*) were concentrated in the early stage of the research period, while emerging journals focusing on simulation teaching and medical informatization (e.g., *Simulation in Healthcare-Journal, JMIR Serious Games*) gained significantly increased influence in recent years, reflecting the trend of research topics toward technicalization and simulation in this field. Figure 4b illustrate co-citation analysis for journals with 30 or more citations, with the most cited journals being *Medical Education, Academic Medicine* and *Medical Teacher*.

**Table 2:** Top-10 journals ranked by publication volume.

Rank	Journal	Publication counts	IF^a^ (2024)
1	BMC medical education	208	3.2
2	Medical teacher	115	4.4
3	Patient education and counseling	60	3.1
4	GMS journal for medical education	49	1.7
5	Journal of medical internet research	45	6.0
6	Academic medicine	45	5.2
7	Medical education	42	5.2
8	Journal of surgical education	37	2.1
9	JMIR medical education	36	12.6
10	Clinical teacher	34	1.2

^a^*IF* Impact factor.

**Table 3:** Top-10 journals ranked by co-citation frequency.

Rank	Journal	Co-citation citations	IF (2024)
1	Medical education	2494	5.2
2	Academic medicine	2342	5.2
3	Medical teacher	2128	4.4
4	BMC medical education	1239	3.2
5	Patient education and counseling	892	3.1
6	Simulation in healthcare	513	2.1
7	JAMA-journal of the American Medical association	502	55.0
8	Journal of medical internet research	482	6.0
9	Advances in health sciences education	447	3.3
10	Journal of general internal medicine	412	4.2

### 3.5 Analysis of references

**Figure 5:**
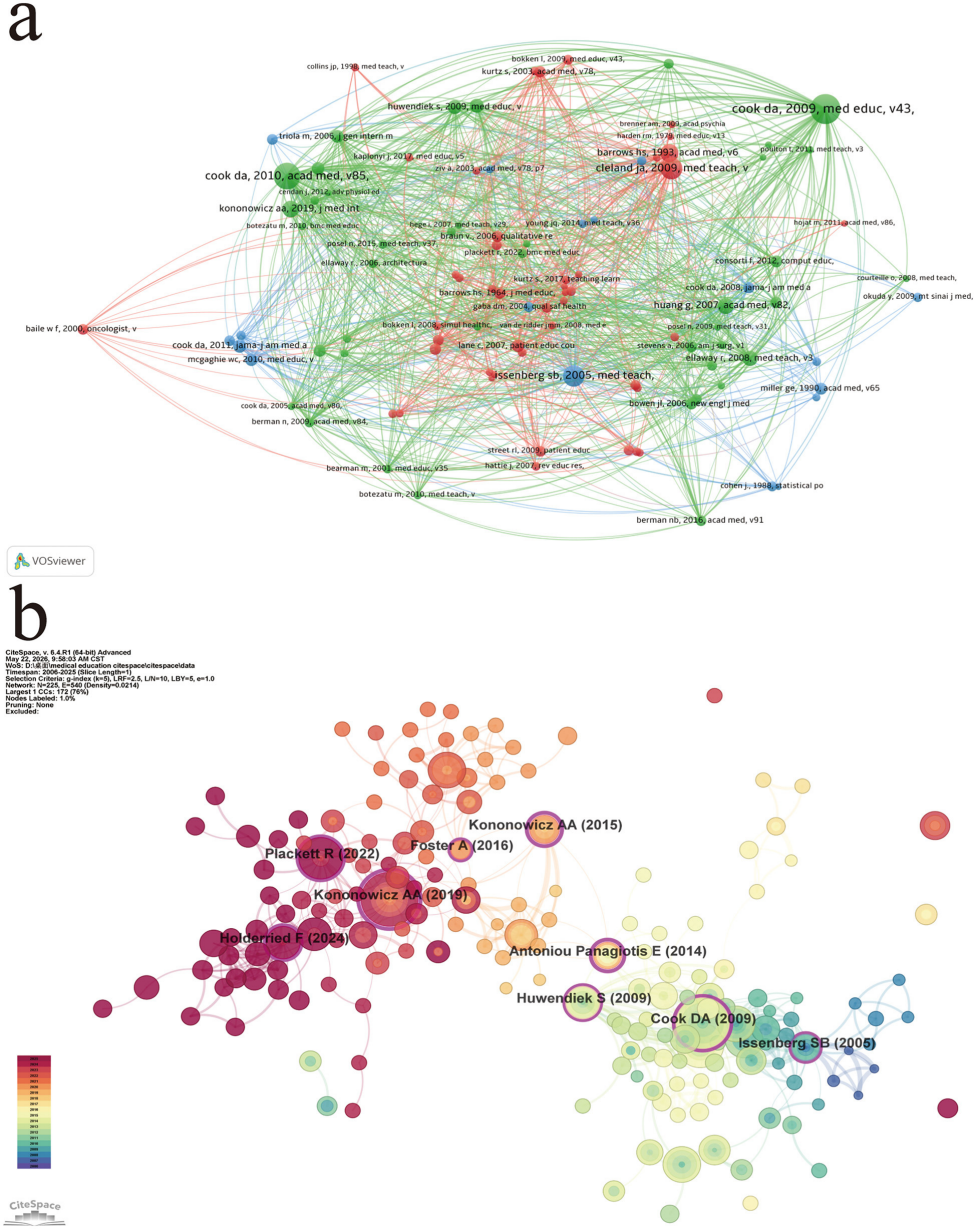
The co-citation analysis of the references related to virtual patients in medical education. **(a)** Map of co-cited references. Node size indicates co-citation frequency, lines show relationships. **(b)** Centrality map of co-cited references. Larger, warmer nodes denote higher betweenness centrality.

**Figure 6:**
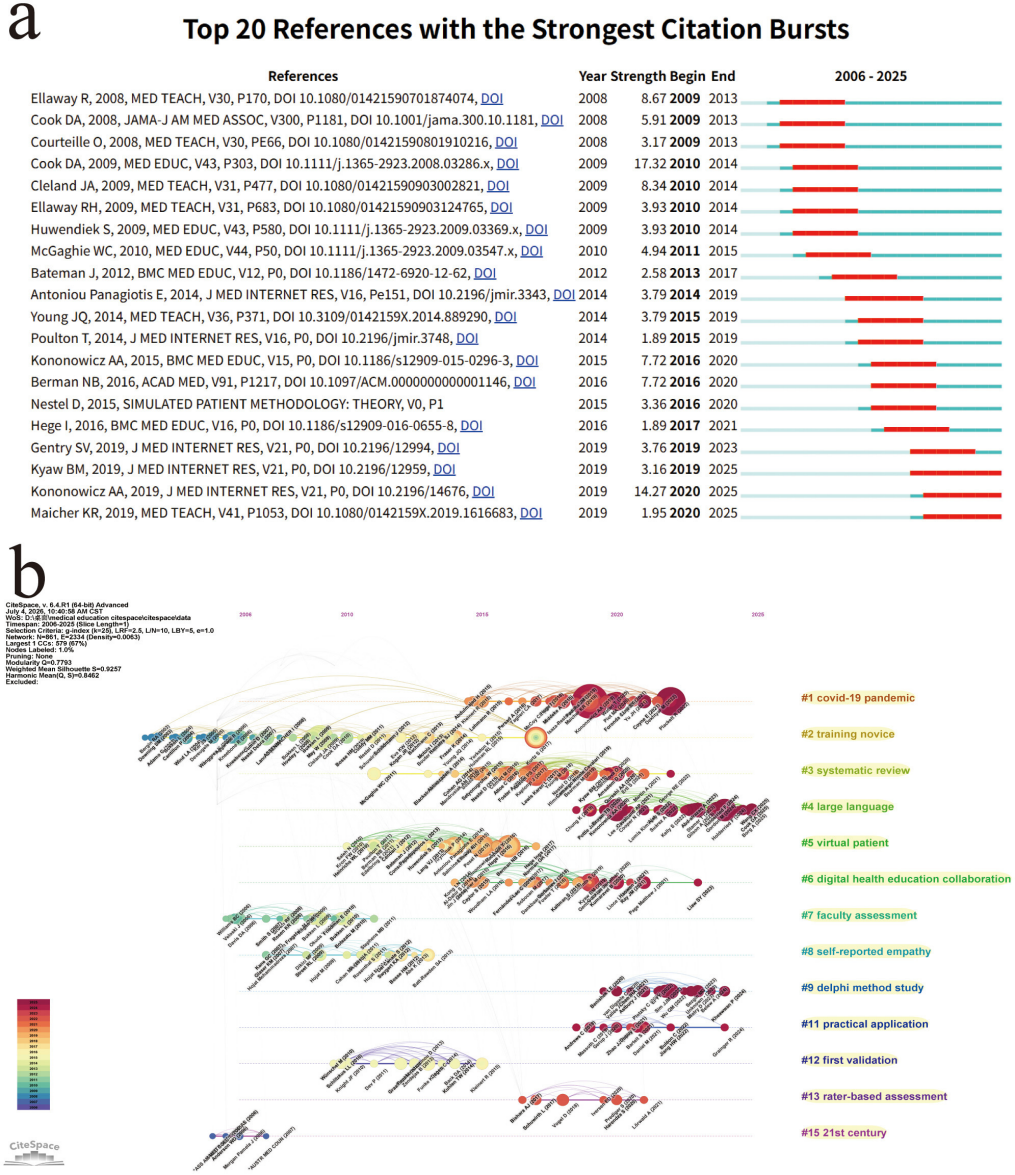
The co-occurrence analysis of the references related to virtual patients in medical education. **(a)** References with the strongest citation burst. “Strength” column shows burst magnitude; colored bars indicate high-citation periods. **(b)** References timeline map. Each horizontal colored line stands for one thematic cluster labeled on the right, nodes represent high-frequency references. The timeline map uses time as the horizontal axis, indicating a continuous growth in publications related to this clustering topic.

After importing the data into VOSviewer and CiteSpace, we obtained the document co-citation map (Figure 5a) and the co-citation centrality map (Figure 5b). Table 4 lists the top five articles with the highest co-citation counts, and Table 5 lists the top five articles with the highest centrality in the past 20 years. The article by Cook, DA.'s team has the highest co-citation count, while the article by Antoniou, PE.'s team has the highest centrality (0.65). Figure 6a selects the 20 articles with the highest citation burst intensity from 2006 to 2025, ranked in descending order of peak citation burst. Figure 6a shows that the article by Cook, DA and colleagues had the highest citation burst intensity (17.32) during 2010–2014, and by the end of 2025, the article by Kononowicz, AA.'s team is expected to reach the highest citation burst intensity.

**Table 4:** Top 5 co-cited references with the highest citation.

Rank	Title	Source	First authors	Year	Citations
1	Virtual patients: a critical literature review and proposed next steps(6)	Medical education	Cook, DA.	2009	120
2	Computerized Virtual Patients in Health Professions Education: A Systematic Review and Meta-Analysis(49)	Medical education	Cook, DA.	2010	99
3	The use of simulated patients in medical education: AMEE Guide No 42(50)	Medical teacher	Cleland, JA.	2009	76
4	Features and uses of high-fidelity medical simulations that lead to effective learning: a BEME systematic review(51)	Medical teacher	Issenberg, SB.	2005	74
5	An overview of the uses of standardized patients for teaching and evaluating clinical skills(52)	Academic medicine	Barrows, HS.	1993	57

**Table 5:** Top 5 co-cited references with the highest centrality.

Rank	Title	Source	First authors	Year	Centrality
1	Exploring design requirements for repurposing dental virtual patients from the web to second life: a focus group study (81)	Journal of medical internet research	Antoniou, PE.	2014	0.59
2	Virtual patient simulations in health professions education: systematic review and meta-analysis by the digital health education collaboration (40)	Journal of medical internet research	Kononowicz, AA.	2019	0.49
3	Virtual patients-what are we talking about? A framework to classify the meanings of the term in healthcare education (82)	BMC medical education	Kononowicz, AA.	2015	0.46
4	Virtual patients: a critical literature review and proposed next steps (6)	Medical education	Cook, DA.	2009	0.37
5	A generative pretrained transformer (GPT)-powered chatbot as a simulated patient to practice history taking: prospective, mixed methods study (47)	JMIR medical education	Holderried, F.	2024	0.18

The document co-citation clustering timeline map (Figure 6b) reveals the thematic structure and temporal evolution trajectory of virtual patient research in medical education. The category with the highest number of publications was cluster 0, so the term “focus group study” was a frequently occurring keyword in this category. Notably, nodes in Cluster 4 “large language models” are predominantly dark red, representing the most recently emerging cutting-edge topic post-2020. In contrast, nodes in Cluster 1 “COVID-19 pandemic” are primarily orange-red, indicating a phase-specific hot topic during the pandemic.

### 3.6 Analysis of keywords

**Figure 7:**
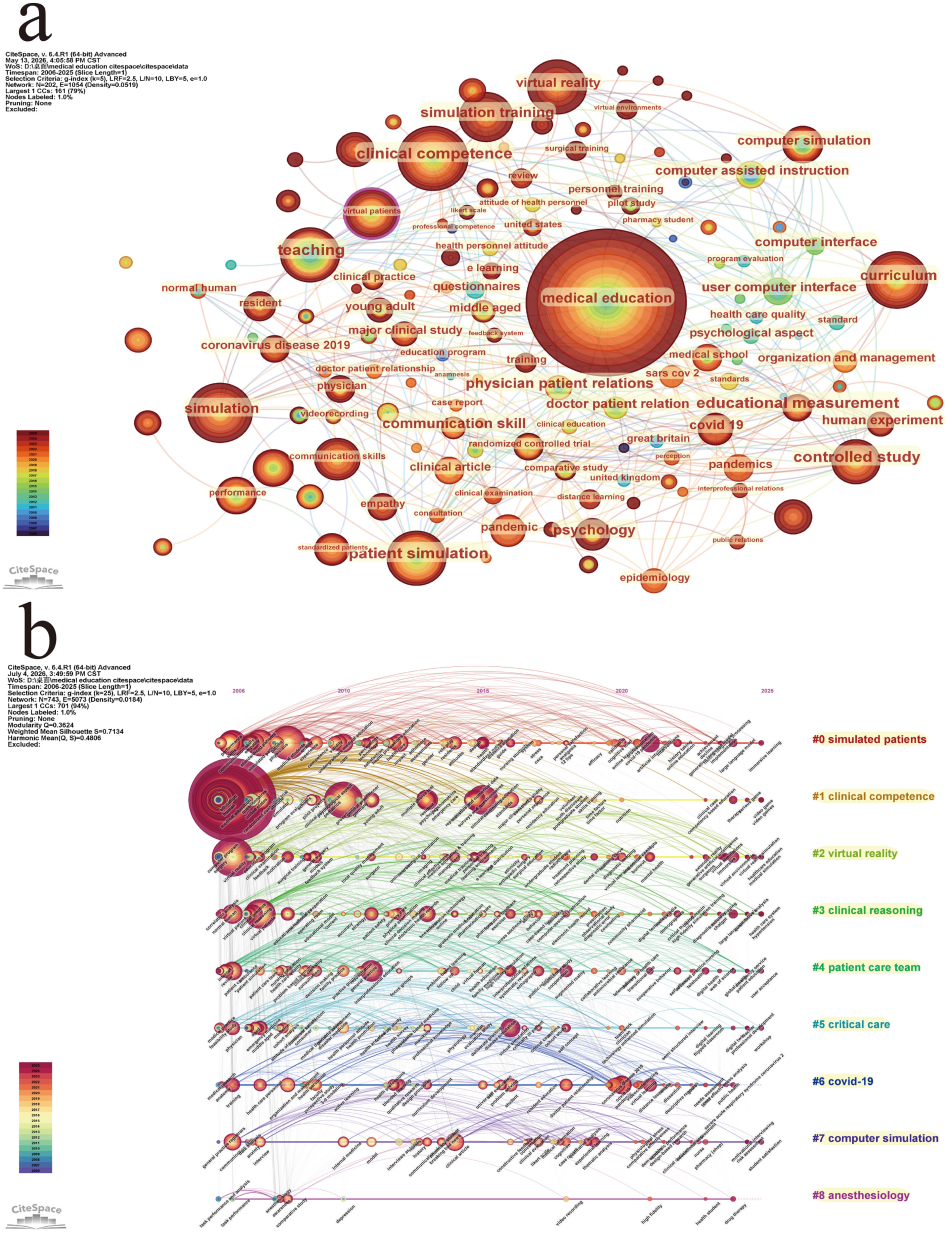
The co-occurrence analysis of the keywords related to virtual patients in medical education. **(a)** CiteSpace network of research keywords. Node rings show publication years, widths indicate annual output, and links represent co-occurrence ties, color gradients mark each term's initial emergence. **(b)** Keywords timeline map. Each horizontal colored line stands for one thematic cluster labeled on the right, nodes represent high-frequency keywords. The timeline map uses time as the horizontal axis, indicating a continuous growth in publications related to this clustering topic.

Table 6 lists the top twenty keywords by frequency over the past 20 years, with “medical education,” “simulation,” “clinical competence,” “virtual reality,” and “patient simulation” appearing most frequently. Figure 7a presents the keyword co-occurrence network of virtual patient research in medical education. Figure 7b presents a timeline view of keywords, displaying nine prominent clusters that visually illustrate the time hotspots and developmental trends in the research field of VPs in medical education. Clusters such as “simulated patients,” “clinical competence,” “virtual reality,” “clinical reasoning,” “patient care team,” “critical care,” “covid-19,” “computer simulation” and “anesthesiology” may emerge as new research hotspots.

**Table 6:** Top 20 keywords with the highest frequency.

Rank	Keywords	Frequency
1	Medical education	1199
2	Simulation	325
3	Clinical competence	318
4	Virtual reality	286
5	Patient simulation	267
6	Curriculum	246
7	Teaching	225
8	Controlled study	175
9	Virtual patients	172
10	Simulation training	168
11	Communication skills	156
12	Skills	147
13	Computer simulation	125
14	Performance	117
15	Educational measurement	117
16	Interprofessional education	101
17	Standardized patients	90
18	Clinical reasoning	82
19	Covid 19	67
20	Artificial intelligence	65

## 4 Discussion

### 4.1 General information

The present bibliometric analysis demonstrates a sustained increase in research on VPs in medical education. However, publication volume alone does not fully capture how the field has evolved in response to external drivers. Our analysis identifies two distinct inflection points along the developmental trajectory of this field: the COVID-19 pandemic (2019–2021) (31, 32) and the rise of GenAI, particularly LLMs (2022–present) (33), each associated with a unique set of evolutionary pathways. The COVID-19 pandemic accelerated the adoption of online and hybrid educational approaches, influencing how learning activities, discussions, and collaborative work were organized. The development of GenAI has also introduced new technological tools that may support ongoing adjustments within the field (34).

The COVID-19 pandemic reshaped research needs in this field. During this period, restrictions on ward teaching and clinical placements limited students' access to experiential clinical learning. In response, VP-based educational approaches were increasingly adopted, positioning VPs as an important tool for maintaining clinical training during periods of educational disruption (35). Although annual publication output did not increase immediately following the outbreak, a marked rise was observed in 2021, which is consistent with the publication lag commonly seen in educational research (36). More importantly, the keyword timeline in Figure 7b reveals the emergence of an independent covid-19 research cluster (cluster #6), while “COVID-19” itself ranks among the top 20 most frequently used keywords (frequency = 67). Figure 4a demonstrates the evolving journal landscape of VPs research in medical education. During the COVID-19 pandemic, digital and simulation education journals including *BMC Medical Education* and *Simulation in Healthcare* saw a continuous rise in the share of VP-related publications and gradually became important publication venues in this field. Collectively, these findings indicate that the pandemic not only stimulated research activity but also contributed to changes in the thematic orientation of VPs research, with increasing attention to remote clinical education, critical care, and technology-enhanced learning (37).

The emergence of GenAI and LLMs has expanded the technological capabilities of VP systems. In contrast to earlier growth drivers, their influence is not primarily reflected in publication volume. “Artificial intelligence” already ranks among the most frequently used keywords (frequency = 65), indicating sustained and growing interest in AI-enhanced VP systems. However, because LLMs only became widely accessible following the release of ChatGPT in late 2022, their bibliometric visibility likely reflects an early-stage technology diffusion phase rather than their actual developmental impact. Instead, their influence is more clearly manifested in a qualitative transformation of VP systems, including natural language interaction, adaptive feedback generation, automated case construction, and enhanced support for clinical reasoning and decision-making (38).

### 4.2 Influential studies and citation dynamics

Co-citation occurs when two or more publications are cited together by subsequent studies. A higher co-citation frequency generally indicates a stronger intellectual relationship between publications and can therefore be used to identify the foundational knowledge base and core themes of a research field (39). Citation bursts, meanwhile, often signal periods of rapid scholarly attention driven by theoretical breakthroughs, methodological innovations, or emerging research frontiers (20).

Table 4 and Table 5 summarize the five most highly co-cited publications and the five publications with the highest betweenness centrality, respectively. Publications with high co-citation frequencies share a focus on educational applications of VPs, and most are review papers or methodological research. Authored by leading researchers and published in high-impact journals, they collectively map the evolution of the field from conceptual development and methodological refinement to technological integration. Consequently, these publications function not only as the intellectual foundations of VPs research but also as pivotal nodes linking multiple research streams and shaping the field's overall knowledge structure. The results presented in Figures 6a, b show that the review published by Cook, DA. et al. (6) is located at the core node of the co-citation network of virtual patient research in medical education. Within the retrieved dataset, this paper has received the highest total citation count and the maximum citation burst value, which reflects its frequent citation by subsequent studies in this field. The review systematically synthesized three core categories of VPs—teaching-focused, problem-solving-focused, and high-fidelity models—and demonstrated the educational effectiveness of VP-based learning across a range of outcomes. Beyond summarizing existing evidence, Cook, DA. et al., proposed a forward-looking research framework centered on five critical dimensions: the educational role of VPs, instructional design and representation, curricular integration, assessment and evaluation, and system development and maintenance. This framework systematically sorts out core research dimensions of VPs, offering clear research directions for subsequent practice and exploration. As shown in the co-citation network (Figures 6a, b), this paper is located at the core node of the dataset and is frequently cited by follow-up studies concerning the design, application, and evolution of VPs in medical education.

The systematic review published by Kononowicz, AA. et al. (40) in 2019 occupies a pivotal position in the contemporary VPs literature, as reflected by its high betweenness centrality (0.49) and strong citation burst (14.27) spanning 2020–2025. Together, these metrics indicate that the study serves as an important bridge between foundational VPs research and more recent developments. The dense “#1 COVID-19 pandemic” cluster in Figure 6b and the increased annual publication output of VPs research after 2020 suggest a growing research attention toward VPs in medical education during the pandemic period. By synthesizing evidence from 51 randomized controlled trials, the review provided strong evidence that VPs can improve clinical skills acquisition among health professions learners while achieving knowledge outcomes that are at least comparable to those associated with conventional educational approaches. Importantly, the study supplied the high-quality evidence needed to support the broader adoption of VP-based education during a period of unprecedented disruption to clinical training. The keyword timeline map (Figure 6b) shows that the #covid 19 cluster clearly reflects the emergence of VP-based teaching as a research hotspot during the pandemic. Notably, the timeline node labeled “cost effectiveness analysis” preceding this cluster further highlights an important practical challenge associated with the implementation of VPs during the pandemic. Although the widespread suspension of face-to-face clinical training made VPs an important alternative instructional modality, their relatively high implementation costs posed a substantial barrier to the broader implementation of VPs in medical education. Whether implemented as conventional screen-based VP systems or immersive simulation platforms, the deployment of VPs generally requires considerable initial investment, including hardware procurement, annual software licensing, and the development of customized clinical cases, together with ongoing maintenance costs. Previous studies have estimated that the development cost of a virtual case is approximately US$12 per student-hour, while the monthly operational cost of a single teaching module can reach US$324.75 (41). During the COVID-19 pandemic, budget constraints experienced by many medical schools limited their capacity to sustain these expenditures over the long term, particularly in resource-limited institutions (42). This economic challenge may partly explain why VPs have not yet achieved widespread implementation in medical education despite their demonstrated educational value after 2020. The financial burden is even greater for immersive VPs training based on virtual reality (VR) or augmented reality (AR), with the one-time procurement cost of a single headset typically ranging from US$2,500 to US$3,000. Moreover, the rapid development of customized interactive cases for emergency remote teaching during the pandemic further increased short-term institutional expenditures (43, 44). Taken together with the temporal trend shown in Figure 6b, the #1 COVID-19 pandemic cluster does not extend to the end of the observation period in the timeline map, suggesting that the surge in research driven by the urgent educational demands of the pandemic has gradually subsided. Nevertheless, the practical challenges highlighted by this cluster—particularly the economic barriers to VP implementation—remain important issues for future research. Addressing the cost of VP deployment is likely to be essential for facilitating their broader and more sustainable integration into medical education.

Meanwhile, emerging GenAI and LLMs may offer a potentially feasible approach to addressing the aforementioned cost barriers. Cook. DA et al. (45) and Li. D et al. have suggested that VP systems driven by GenAI and LLMs are relatively low-cost, reusable, and capable of automatically generating clinical cases, thereby reducing the time and human resources required for case development while increasing the diversity of training scenarios (46). In Figure 6b, the “large language model” cluster 4 is highlighted in dark red, with densely connected nodes and a concentration of recent publications on the right side of the timeline (within the past 5 years). This pattern reflects a growing body of literature in recent years focusing on the integration of LLMs into virtual patient systems, indicating an increasing research interest in this area. Of particular note, the 2024 study by Holderried F. et al. (47) ranks among the top five publications in terms of betweenness centrality for all VP-related literature between 2006 and 2025, reflecting the increasing research attention devoted to GenAI integration. The study applied a GPT-based model to a VP system designed for clinical consultation training. The system does not require continuous involvement from clinical instructors or standardized patients (SPs), and it eliminates the need for extensive human labor associated with one-to-one guidance and manual review of individual dialogues. This enables medical students to engage in self-directed, repeated practice without temporal or spatial constraints and to receive immediate feedback, thereby increasing the frequency of clinical interviewing practice and improving teaching throughput efficiency. This study provides important evidence supporting the potential of GenAI to optimize VPs systems. In addition, a multicenter randomized controlled trial further demonstrated that AI-based VPs training can reduce per-student training costs by nearly 40% compared with standardized patient programs, while enabling unlimited repetitive practice and delivering real-time instructional feedback comparable to that of professional tutors (48). These cost advantages and scalability suggest that GenAI-enabled VPs may represent a promising approach for addressing financial constraints and promoting the broader adoption of virtual simulation tools in the post-pandemic era.

Overall, the early high-co-citation literature in the field was dominated by systematic reviews, conceptual studies, and technology-oriented investigations. These studies primarily focused on the educational evaluation, effectiveness, and future development of VPs in medical education, offering systematic theoretical foundation and clear research directions for subsequent studies (49–52). Among these high-co-citation papers, additional large-scale meta-analyses conducted by Cook, DA. et al. (49) further demonstrated that simulation-based interventions outperformed training without simulated practice, shifting research focus from the necessity of using VPs to strategies for optimizing their educational application (53, 54). Driven by the widespread disruption of in-person clinical training, research on VPs received increasing scholarly attention during the COVID-19 pandemic. Medical schools in many settings across institutions increasingly adopted VPs resources to compensate for the loss of experiential learning opportunities caused by restricted clinical rotations and limited access to real patient encounters. High-fidelity virtual simulation systems thus served as an emergency alternative in educational settings in multiple regions (55, 56). Although the number of studies in this area has continued to increase across different regions, the broader implementation of VPs training tools remains limited, partly due to their high development and deployment costs (57). Furthermore, bibliometric findings together with representative high-impact studies suggest that, in the post-pandemic context, blended learning models have shown advantages over traditional face-to-face instruction in terms of student knowledge acquisition (58). Our bibliometric analysis of this field shows that VP-related scholarly papers increasingly explore combinations of VPs and GenAI/LLMs, reflecting a shift in research focus from traditional standalone simulation frameworks toward digital learning prototypes embedded with generative AI. Within existing academic trials, LLMs have been tested to act as virtual patient avatars, customized tutoring modules and automatic teaching content generators. Several experimental LLM-integrated VP prototypes demonstrated performance exceeding that of junior medical trainees on specific medical knowledge assessments conducted under controlled research settings (59). In addition, LLM-enabled VPs are capable of delivering standardized, personalized, and real-time instructional feedback. This may effectively enhance medical students' clinical reasoning and decision-making skills, while creating training workflows that more closely resemble real clinical scenarios (60, 61). Leveraging the core advantages of automated case generation and low-cost, lightweight deployment enabled by LLMs, such intelligent VP systems have the potential to reduce the high development and operational costs associated with traditional simulation platforms. This may help alleviate long-standing financial constraints that have limited the broader adoption of VP systems.

### 4.3 Shifting themes and emerging technologies

Keywords provide concise representations of an article's main topics and research focus, encapsulating its themes, ideas, essence, as well as its hotspots and trends (62). Analyzing these aspects helps identify research frontiers and major areas of focus of research in a given field (63). Keyword analyses suggest that VPs research has gradually expanded from early educational applications toward broader functions involving clinical assessment, feedback, and technology-enhanced learning. As shown in Figure 7a, b, and Table 6, clusters such as #0 simulated patients, #1 clinical competence, #2 virtual reality, #3 clinical reasoning, #6 COVID-19, and #7 computer simulation, along with high-frequency keywords including “clinical competence,” “virtual reality,” “curriculum,” “teaching,” “simulation training,” “communication skills,” “interprofessional education,” “standardized patients,” “clinical reasoning,” “COVID-19,” and “artificial intelligence,” collectively reflect the evolution of research priorities.

Simulation-based medical education initially relied primarily on SPs, who are trained human actors used for history-taking, communication, and clinical skills training (64, 65). Although SPs differ fundamentally from computer-based VPs, they provide important historical context for understanding the evolution of simulation-based medical education. During this early stage, Objective Structured Clinical Examinations (OSCEs) commonly used SPs to assess clinical performance (66). With advances in digital educational technologies, computer-based VPs were gradually introduced into medical curricula. Comparative studies subsequently demonstrated that VPs achieved learning outcomes comparable to those of SPs (67). Furthermore, a 2010 meta-analysis provided robust evidence that computer-based VPs significantly improved medical students' clinical skills, supporting their broader adoption in medical education (49).

Another major theme concerns the application of VPs in clinical reasoning and skills development. VPs have increasingly been developed as interactive platforms that provide safe and repeatable simulated clinical pathways, and have been applied across diverse settings, including perioperative surgical training (68), material selection in dental education (69), and veterinary and basic sciences (70). These platforms not only enhance students' self-efficacy and reasoning abilities but also support interprofessional education (IPE) by fostering collaborative learning and integrated patient care. Moreover, by overcoming temporal and spatial constraints, VPs have increasingly been incorporated into blended learning models, serving as effective and flexible educational tools (71).

In recent years, the integration of GenAI, LLMs, VR, and XR has introduced new thematic directions. As shown in the timeline cluster map in Figure 7b, the keyword “large language models” appears within the temporal nodes of the #3 clinical reasoning cluster. This suggests that research on GenAI and LLM-enabled virtual patients has extended to the domain of clinical reasoning training, a core component of medical education, and is increasingly being explored as a potential technological approach to support the development of medical students' clinical practice competencies. Current research focuses on GenAI-driven real-time interactions and adaptive feedback derived from electronic health records, which may improve the authenticity and scalability of training in history-taking, clinical reasoning, and decision-making (61, 72). Within the #0 simulated patient cluster, the presence of the keyword “immersive learning” at the rightmost end of the timeline further indicates another emerging research focus in recent years. Immersive multi-user environments provide low-cost, repeatable, and risk-free settings for practicing complex clinical skills, including interventional procedure planning and mass-casualty triage in emergency medicine (73, 74). VPs are increasingly recognized as more than supplementary teaching tools and have been explored as important components of medical training (75), with learners reporting greater engagement with the VPs format (76). Beyond knowledge delivery, VP applications have expanded to cultivate higher-order professional competencies, such as empathy, stress management in breaking bad news, and ethical decision-making with diverse patient populations (77, 78). Nevertheless, challenges persist, including the unpredictability of GenAI outputs and limitations in replicating the depth of human dialogue (79). Despite these concerns, empirical evidence demonstrates significant gains in overall clinical competence, as reflected in improved assessment scores. Consequently, VPs continue to represent an important and evolving component of post-pandemic medical education (80).

## 5 Limitation

All literature records analyzed in this bibliometric study were extracted exclusively from the WOS Core Collection and Scopus databases. The rigorous screening and indexing criteria of these two mainstream academic databases help guarantee the overall reliability of our analytical results. Nevertheless, this research still has several inherent limitations that should be acknowledged objectively.

First, the study adopted a fixed retrieval time window from 2006 to 2025, does not capture literature published before 2006; the limited time span may hinder a full tracing of the earliest embryonic stage of virtual patient research in medical education.

Second, database selection bias may exist, as both WOS and Scopus primarily index journals with international visibility, while regionally indexed or discipline-specific journals may be excluded. This may affect the comprehensiveness of the global research landscape and collaboration patterns.

Third, obvious language bias exists in the included literature. WOS and Scopus predominantly index English-language academic outputs, while high-quality research published in non-English languages (such as Chinese, Spanish, French and German) is not fully covered, which may skew the distribution characteristics of global research cooperation and hotspots.

Fourth, only peer-reviewed journal articles indexed by the two databases were included, and unindexed gray literature including conference preprints, local institutional reports, unpublished master's and doctoral dissertations, and regional educational research bulletins were excluded. Relevant valuable research may be omitted, which cannot be fully reflected in the bibliometric network and trend analysis.

Fifth, citation bias should be considered when interpreting the results. Bibliometric indicators are influenced by cumulative citation advantages (e.g., the Matthew effect), whereby highly cited studies may disproportionately shape perceived research impact, independent of their educational or clinical relevance.

Sixth, bibliometric visualization tools (CiteSpace and VOSviewer) rely on citation and co-occurrence relationships built by indexed metadata, which cannot incorporate the subjective clinical value and practical educational effect of each virtual patient study.

Finally, it is important to distinguish between publication productivity and scientific or educational impact. A higher publication volume does not necessarily indicate greater scientific influence or educational effectiveness. While bibliometric indicators such as citations and keyword co-occurrence provide useful insights into research trends, they remain limited in capturing the real-world educational value of virtual patient applications.

Despite these limitations, this study provides a comprehensive overview of the evolution of virtual patient research in medical education and offers valuable insights into its thematic and structural development.

## 6 Conclusion

This study conducted a bibliometric analysis of research on VPs in medical education from 2006 to 2025. The findings identified the most influential countries, institutions, journals, and references within the field, as well as key research hotspots, primary focus areas, and emerging directions for future investigation. Driven by the continued advancement of GenAI and LLMs, medical education continues to evolve alongside advances in interdisciplinary collaboration and digital technologies. VPs have evolved from auxiliary teaching tools into important components of contemporary medical curricula.

Looking ahead, future development of GenAI and LLM-generated VPs should address challenges such as the risk of medical inaccuracies due to GenAI hallucinations and the current limitations in simulating non-verbal clinical cues. Overcoming these challenges will enable the development of more dynamic, complex, and precise simulated scenarios, better equipping medical students to manage the multifaceted realities of patient care. Enhancing the effectiveness of VPs is expected to provide educators and learners with more efficient and impactful medical teaching and learning experiences.

## Data Availability

The original contributions presented in the study are included in the article/Supplementary material, further inquiries can be directed to the corresponding author/s.
