# Supplementary material for: Virtual patients in medical education: a bibliometric analysis based on the wos core collection and scopus databases
Source: Front Med (Lausanne). 2026 Jul 30;13:1904001. doi: 10.3389/fmed.2026.1904001 (PMC13470259; doi:10.3389/fmed.2026.1904001)
Supplement: Supplementary file 1 [file Table_1.docx]

Supplementary Material for “Virtual Patients in Medical Education: a Bibliometric and Visualization Analysis”

**Contents**

1.Supplementary Tables

2.Supplementary Figures

1. Supplementary Tables

**Supplementary Table 1.** A detailed description of the search terms and strategy.

| **Source and coverage** | **Search string** | | **Results** | **Notes** |
| --- | --- | --- | --- | --- |
| Scopus (Elsevier)  Coverage:  From database inception-2025-12-31 | ( TITLE-ABS-KEY ( "virtual patient*" OR "simulated patient*" OR "digital patient*" OR "virtual simulation" OR "virtual case*" OR "virtual environment" OR "computer based patient*" ) AND TITLE-ABS-KEY ( "Medical Education" OR "Clinical Skills Training" OR "Health Professions Education" OR "medical education research" OR "Interprofessional Education" OR "IPE" OR "Undergraduate Medical Education" OR "Continuing Medical Education" OR "medical student*" OR "medical resident*" ) ) AND PUBYEAR > 2005 AND PUBYEAR < 2026 AND ( LIMIT-TO ( DOCTYPE , "ar" ) OR LIMIT-TO ( DOCTYPE , "re" ) ) AND ( LIMIT-TO ( LANGUAGE , "English" ) ) | | 2100 results | All search terms are searched in the fields: “title”, “abstract” and “keywords” (here marked with “TITLE-ABS-KEY”) filters or limitations English language, articles and reviews  Years 2006-2025 |
| Web of Science-Core Collection (Clarivate)  Coverage:  From database inception-2025-12-31 | #1 "virtual patient*" OR "simulated patient*" OR "digital patient*" OR "virtual simulation" OR "virtual case*" OR "virtual environment" OR "computer based patient*" (Topic)  <http://webofscience.clarivate.cn/wos/woscc/summary/66272f35-d7c7-4589-8324-35e2404e924c-01b242d95e/relevance/1>  #2"Medical Education" OR "Clinical Skills Training" OR "Health Professions Education" OR "medical education research" OR "Interprofessional Education" OR "IPE" OR "Undergraduate Medical Education" OR "Continuing Medical Education" OR "medical student*" OR "medical resident*"  <http://webofscience.clarivate.cn/wos/woscc/summary/732efc78-8882-4b5f-8aac-4e666835e45b-01b242df69/relevance/1>  #1 AND #2  <http://webofscience.clarivate.cn/wos/woscc/summary/0d4f10a9-422b-4818-8e07-c471d6cd32a7-01b242e86b/relevance/1> | | 1113 results | All search terms are searched in the field: “Topic” (including title, abstract and author supplied keywords, here marked with “TOPIC”). filters or limitations English language, articles and reviews  Years 2006-2025 |
| Total no. references identified | | 3213 |  |  |
| Total no. unique references identified after automatic de-duplication in RStudio and CiteSpace | | 2347 |  |  |
| duplicate | | 866 |  |  |
| Total no. references identified | | 3213 |  |  |

**Supplementary Table 2.** Top 10 authors ranked by publication output.

| **Rank** | **Author** | **Number of Documents** |
| --- | --- | --- |
| 1 | Harendza, S. | 22 |
| 2 | Hege, I. | 20 |
| 3 | Nestel, D. | 20 |
| 4 | Rethans, JJ. | 19 |
| 5 | Zary, N. | 17 |
| 6 | Edelbring, S. | 15 |
| 7 | Fors, U. | 15 |
| 8 | Lok, B. | 15 |
| 9 | Raupach, T. | 13 |
| 10 | Lajoie, SP. | 12 |

**Supplementary Table 3.** Top 10 authors ranked by total citation frequency.

| **Rank** | **Author** | **Total Citations** |
| --- | --- | --- |
| 1 | Cook, DA. | 512 |
| 2 | Hojat, M. | 202 |
| 3 | Barrows, HS. | 166 |
| 4 | Kononowicz, AA. | 132 |
| 5 | Llaway, R. | 130 |
| 6 | Huwendiek, S. | 128 |
| 7 | Issenberg, SB. | 121 |
| 8 | Bokken, I. | 121 |
| 9 | Triola, MM. | 118 |
| 10 | Nestel, D. | 117 |

2.Supplementary Figures

**Fig. S1** Collaborative network among authors. (a) Analysis of collaborative network visualization of authors in VOSviewer. Node size denotes frequency of the authors' appearance, line thickness indicates inter-author academic relevance (b) A visualization map of authors with ≥ 30 citations. Node size represents the co-citation frequency of authors, line strength reflects the academic relevance between authors.
